# Aspartate metabolic flux promotes nitric oxide to eliminate both antibiotic-sensitive and -resistant *Edwardsiella tarda* in zebrafish

**DOI:** 10.3389/fimmu.2023.1277281

**Published:** 2023-10-11

**Authors:** Jiao Xiang, Min-yi Li, Hui Li

**Affiliations:** ^1^ State Key Laboratory of Bio-Control, School of Life Sciences, Sun Yat-sen University, Guangzhou, China; ^2^ Laboratory for Marine Fisheries Science and Food Production Processes, Qingdao National Laboratory for Marine Science and Technology, Qingdao, China; ^3^ Guangdong Province Key Laboratory for Pharmaceutical Functional Genes, Sun Yat-sen University, Guangzhou, China; ^4^ Southern Marine Science and Engineering Guangdong Laboratory (Zhuhai), Sun Yat-sen University, Guangzhou, China

**Keywords:** *Edwardsiella tarda*, aspartate, nitric oxide, reprogramming metabolome, antibiotic-free approach, zebrafish, sodium nitroprusside

## Abstract

**Introduction:**

Metabolic reprogramming potentiates host protection against antibiotic-sensitive or -resistant bacteria. However, it remains unclear whether a single reprogramming metabolite is effective enough to combat both antibiotic-sensitive and -resistant bacteria. This knowledge is key for implementing an antibiotic-free approach.

**Methods:**

The reprogramming metabolome approach was adopted to characterize the metabolic state of zebrafish infected with tetracycline-sensitive and -resistant *Edwardsiella tarda* and to identify overlapping depressed metabolite in dying zebrafish as a reprogramming metabolite.

**Results:**

Aspartate was identify overlapping depressed metabolite in dying zebrafish as a reprogramming metabolite. Exogenous aspartate protects zebrafish against infection caused by tetracycline-sensitive and -resistant *E. tarda*. Mechanistically, exogenous aspartate promotes nitric oxide (NO) biosynthesis. NO is a well-documented factor of promoting innate immunity against bacteria, but whether it can play a role in eliminating both tetracycline-sensitive and -resistant *E. tarda* is unknown. Thus, in this study, aspartate was replaced with sodium nitroprusside to provide NO, which led to similar aspartate-induced protection against tetracycline-sensitive and -resistant *E. tarda*.

**Discussion:**

These findings support the conclusion that aspartate plays an important protective role through NO against both types of *E. tarda*. Importantly, we found that tetracycline-sensitive and -resistant *E. tarda* are sensitive to NO. Therefore, aspartate is an effective reprogramming metabolite that allows implementation of an antibiotic-free approach against bacterial pathogens.

## 1 Introduction

*Edwardsiella tarda* is a Gram-negative bacterium that causes infection in aquatic animals and humans, posing a threat to aquaculture industry and public health (1, 2). Antibiotics are effective against bacteria, but the extensive use of these drugs leads to the development of antibiotic resistance due to the consequent selective pressure. Bacterial strains that are resistant to antibiotics are difficult to treat and the increasing emergence of these strains limits sustainable development of aquaculture. Given that overuse of antibiotics triggers the development of antibiotic-resistant bacteria (3), antibiotic-free approaches are especially recommended to eliminate bacterial pathogens (4).

Reprogramming metabolomics is a new useful approach for controlling bacterial infection by using low doses of antibiotics or no antibiotics at all (5–11). This method requires the use of comparative metabolomics to identify crucial biomarkers, which are then used to induce metabolome-reprogramming for elevating bacterial sensitivity to antibiotics or improving host protection against infection (12–16). This leads to subsequent host protection against infections caused by antibiotic-sensitive or –resistant *Vibrio alginolyticus* and *Edwardsiella tarda* (17–21). However, the question of whether a single reprogramming metabolite can be used to combat both antibiotic-sensitive and –resistant bacteria has not been well defined. Importantly, antibiotic-free therapy is required to effectively combat both sensitive and resistant bacteria. This success will help in the development of antibiotic-free methods to eliminate bacterial pathogens.

Among antibiotic classes used in aquaculture, tetracyclines, including tetracycline and oxytetracycline, are the first-line agents used for treatment. As a result, there have been increasing incidences of tetracycline-resistant *E. tarda*. Lo et al., showed that 21.3% (20/94) of isolates were resistant to oxytetracycline (22). Further, Yu et al., isolated *E. tarda* CK41 from Japanese flounder diagnosed with edwardsiellosis, which is highly resistant to multiple antibiotics, including tetracycline (23). Lee and Wendy showed that out of 300 *E. tarda* and *Aeromonas hydrophila* strains, 58% were tetracycline-resistant (24). However, the reprogramming metabolomics approach has not yet been utilized to combat tetracycline-resistant bacteria. Importantly, for successful implementation of an antibiotic-free approach, reprogramming of a single metabolite must be effective for both antibiotic-sensitive and -resistant bacteria, but this has not yet been reported in the literature. Therefore, we sought to use tetracycline-resistant and -sensitive *E. tarda* may as representative bacterial strains to identify one reprogramming metabolite to eliminate both strains.

Herein, we used gas chromatography-mass spectrometry (GC-MS) based metabolomics to characterize differential metabolic profiles of zebrafish infected with a tetracycline-resistant strain (LTB4-R_TET_) and -sensitive strain (LTB4-S). All data were compared to uninfected control animals. In this way, we sought to identify an overlapping biomarker between LTB4-R_TET_ and LTB4-S that could potentially be exploited as a reprogramming metabolite to improve host protection against both antibiotic-sensitive and –resistant *E. tarda*. Following data analysis, we identified aspartate as a crucial biomarker and subsequently used it to reverse the infective phenotype in zebrafish, thereby increasing survival.

## 2 Materials and methods

### 2.1 Ethics statement

The study was approved by the Institutional Animal Care and Use Committee of Sun Yat-sen University, Guangzhou, China.

### 2.2 Animals and bacterial strains

Zebrafish (*Danio rerio*), approximately 3 months old (body length, 3 ± 0.2 cm, body weight, 0.2 ± 0.05 g), were purchased from Guangdong Zebrafish Breeding Farm (Guangzhou, China). Zebrafish were acclimated for 2 weeks in 540 L water tanks with Closed Recirculating Aquaculture Systems. In the meantime, fish were fed twice a day, and water tanks were cleaned by siphoning the food debris and feces once every two days. Before experiments, all fish were tested to ensure they were not infected with *E. tarda*. *E. tarda* LTB4-S and LTB4-R_TET_ were preserved in our laboratory. After a single colony was cultured for 24 h at 30°C, with shaking at 200 rpm in fresh 50 mL TSB medium, the bacterial suspensions were diluted 1:100 in fresh TSB medium cultured and grown to an optical density at 600 nm (OD_600_) of 1.0 at 30°C.

### 2.3 Supplementation of exogenous metabolites and bacterial challenge

Exogenous metabolites were supplemented as previously described (25). Zebrafish were challenged with 1 × 10^5^ CFU/fish. Then each zebrafish were intraperitoneally injected with 5 μL of a 12 μg/μL suspension of aspartate (60 μg total dose) or 0.75 ~ 6 μg sodium nitroprusside dissolved in saline for 3 days, once a day. Control fish were injected with 5 μL saline solution. After 30 h, humoral fluid was collected for GC-MS analysis and spleen samples were collected for qRT-PCR, nitric oxide synthase (NOS) activity, and NO content detection.

### 2.4 Measurement of bacterial content in zebrafish by quantitative polymerase chain reaction

Next, qPCR was adopted to quantitate *E. tarda* in zebrafish as described previously (26). In brief, bacteria (10^3^, 10^4^, 10^5^, 10^6^, 10^7^, and 10^8^ CFU) were used to extract bacterial genomes. PCRs with primers for the *gyrB* gene were used to assess bacterial load along a standard curve. The same numbers of LTB4-S and LTB4-R_TET_ (5 × 10^3^ CFU) were intraperitoneally injected into zebrafish. The zebrafish that survived for 6–144 h were collected. The HiPure Tissue DNA Mini Kit (Magen Biotechnology, Guangzhou, China) was then used to extract genomic DNA and bacterial DNA was subsequently measured by qPCR. The amount of bacterial DNA was then calculated based on the aforementioned standard curve.

### 2.5 Measurement of minimum inhibitory concentration and growth curve.

MICs of LTB4-S and LTB4-R_TET_ were measured by antimicrobial susceptibility testing as described in Clinical & Laboratory Standards Institute guidelines (27). The bacterial growth curve was determined according to conventional procedures.

### 2.6 Sample preparation for GC-MS analysis

Samples were prepared as previously described (28). In brief, LTB4-S and LTB4-R_TET_ were cultured in LB medium and collected by centrifuged to infect zebrafish using 1 × 10^5^ CFU/fish. Dying zebrafish were collected, rinsed and wiped. These zebrafish were cut into six pieces, weighted, and added appropriate volume of saline (100 μL/100 mg) for humoral. After centrifugation, 50 μL supernatant was collected for metabolite extraction using precooled methanol. Following centrifugation, supernatant was collected and then dried by vacuum centrifuge device for GC-MS analysis.

### 2.7 Analysis of metabolomic data

Analysis was performed according to known methods (29). In brief, XCalibur software was used to analyze the mass fragmentation spectrum. The National Institute of Standards and Technology (NIST) library and NIST MS search 2.0 program were adopted to match the data to identify compounds. Software IBM SPSS Statistics 19 was used to analyze significant difference of the standardized data, when the differences were defined at P value < 0.05. SIMCA-P + 12.0 software were used to perform principal-component analysis (PCA) and S-plot analysis. iPath3.0 (https://pathways.embl.de/) was used to carry out interactive Pathways (iPath) analysis.

### 2.8 Quantitative reverse transcription PCR

Next, qRT-PCR was performed according to previously published methods (30). Gene-specific primers that were used here are shown in **Table 1**. The β-actin gene served as the internal control.

**Table 1:** Primers used for qRT-PCR.

Genes	Primer	Sequence (5’-3’)
*β-actin*	Forward	ACCCAGACATCAGGGAGTG
	Reverse	CATCCCAGTTGGTCACAATAC
*ass1*	Forward	GGCATTCTGGAGAACCCCAA
	Reverse	CAGAAAATCTCCAGCGGGGT
*as1*	Forward	TTGCTGGGAATCCCTTCGAC
	Reverse	TGCCATCTTGCTAAGGTGTGT
*arg2*	Forward	GCCATTCTCAGCAGTGTCCT
	Reverse	AATCCGGGAACTTTGGGCAT
*nos2a*	Forward	TGCAATCACTGTGTTCCCTCA
	Reverse	AGCACATCAAAGCGACCGTA
*nos2b*	Forward	GTGCTGGAGGAGTTTCCCTC
	Reverse	GAGGTCAGGAGAGGAGCTGA
*gyrB*	Forward	GACGGCGGGACCCATTT
	Reverse	CGGCACCTTCACGGACA

### 2.9 Measurement of NOS activity/NO content

A series of experiments were performed according to kit instructions (Nanjing Jiancheng Bioengineering Institute, Nanjing, China). The spleens from ten zebrafish were pooled into one biological sample and three biological replicates were measured per group.

## 3 Results

### 3.1 Phenotypes of LTB4-R_TET_ and LTB4-S and their survival in zebrafish

**Figure 1:**
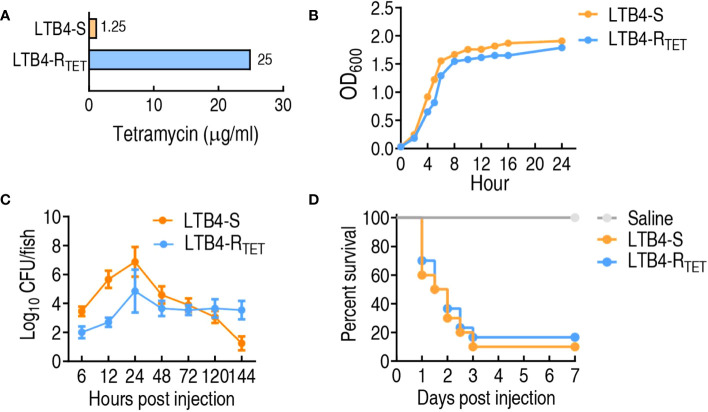
Resistance and growth phenotypes of LTB4-S and LTB4-R_TET._ **(A)** Minimum inhibitory concentration of LTB4-S and LTB4-R_TET_ to tetracycline. **(B)** LTB4-S and LTB4-R_TET_ growth curve. **(C)** Dynamic changes in bacterial number in zebrafish infected with LTB4-S and LTB4-R_TET._ **(D)** Survival of zebrafish infected with LTB4-S and LTB4-R_TET_.

LTB4-R_TET_ and LTB4-S were obtained through sequential propagation of LTB4 in medium with and without tetracycline, respectively. This led to the revelation that LTB4-R_TET_ has a 20-fold higher MIC for tetracycline compared to that of LTB4-S, which showed no change in the MIC during propagation (**Figure 1A**). Growth curves showed that LTB4-R_TET_ proliferated more slowly than LTB4-S (**Figure 1B**). When zebrafish were infected with LTB4-S or LTB4-R_TET,_ the two strains differentially persisted in the zebrafish. Specifically, more LTB4-S were detected in the fish during the 6–48 h time points compared to LTB4-R_TET_. Further, these bacteria were measured again at the 72, 120, and 144 h time points and we identified reduced numbers of LTB4-R_TET_ compared to LTB4-S at the latter (**Figure 1C**). Similar survival rates were determined for LTB4-R_TET_ and LTB4-S infected zebrafish (**Figure 1D**). Therefore, we concluded that both LTB4-R_TET_ and LTB4-S infection causes differential growth rates and resistance phenotypes, but similar survival rates.

### 3.2 Metabolic profiles in dying zebrafish infected with LTB4-R_TET_ and LTB4-S.

**Figure 2:**
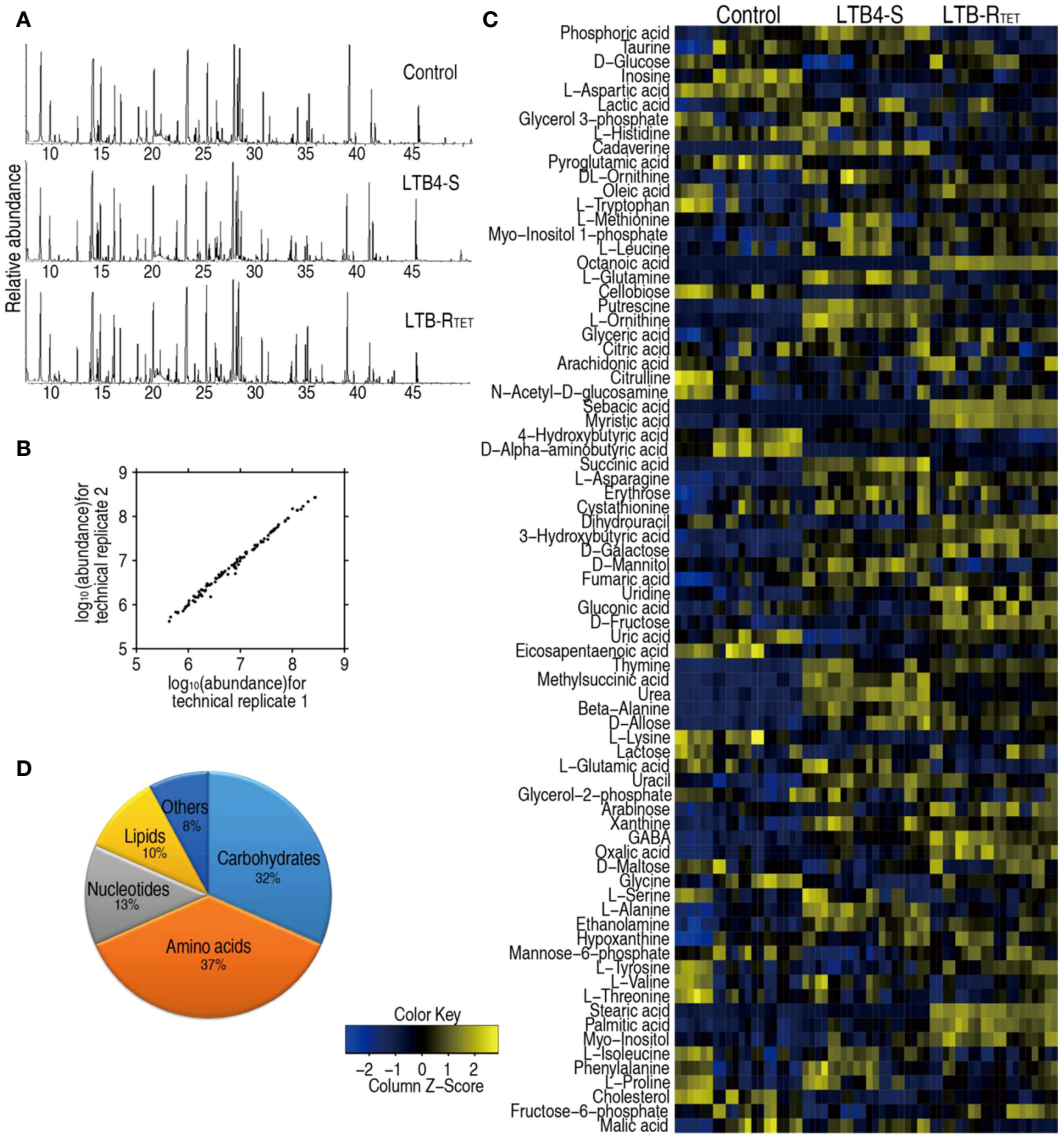
Metabolic profiles of LTB4-S and LTB4-R. **(A)** Samples of total ion current chromatogram separately from control uninfected animals and LTB4-S and LTB4-R_TET_-infected animals. **(B)** Evaluation for reproducibility of the metabolomic profiling platform. Pearson correlation coefficient of metabolite abundance between technical replicates varies between 0.994 and 0.999. **(C)** Heat map showing unsupervised hierarchical clustering by using metabolites (row). Blue, downregulated; yellow, upregulated (see color scale). **(D)** Categories of all metabolites by searching against KEGG.

Host metabolic state is correlated with susceptibility or resistance to bacterial pathogens (29, 31). Thus, the metabolic profiles of zebrafish infected with LTB4-R_TET_ and LTB4-S were compared and uninfected control fish. To do this, a GC-MS-based metabolomics approach was utilized to characterize the metabolic profiles of dying animals in the two experimental groups and in the control group. Ten biological samples with two technical replicates were adopted for each group, yielding a total of 60 data sets. A total of 230 aligned individual peaks were obtained from each sample, where 77 metabolites were determined (**Figure 2A**). The correlation coefficient for technical replicates varied between 0.994 and 0.999 (**Figure 2B**), suggesting good repeatability of the data. Metabolic profiles of these 77 metabolites are displayed as a heatmap for each group, which shows that for each group, LTB4-S and LTB4-R_TET_ infected and uninfected zebrafish clustered independently (**Figure 2C**). According to the KEGG annotation, the identified metabolites were classified as 37% amino acids, 32% carbohydrate, 13% nucleotide, 10% lipid, and 8% as other (**Figure 2D**). These data suggest that this metabolic platform provides reliable data for further analysis.

### 3.3 Differential metabolic profiles in zebrafish infected with LTB4-R_TET_ and LTB4-S

**Figure 3:**
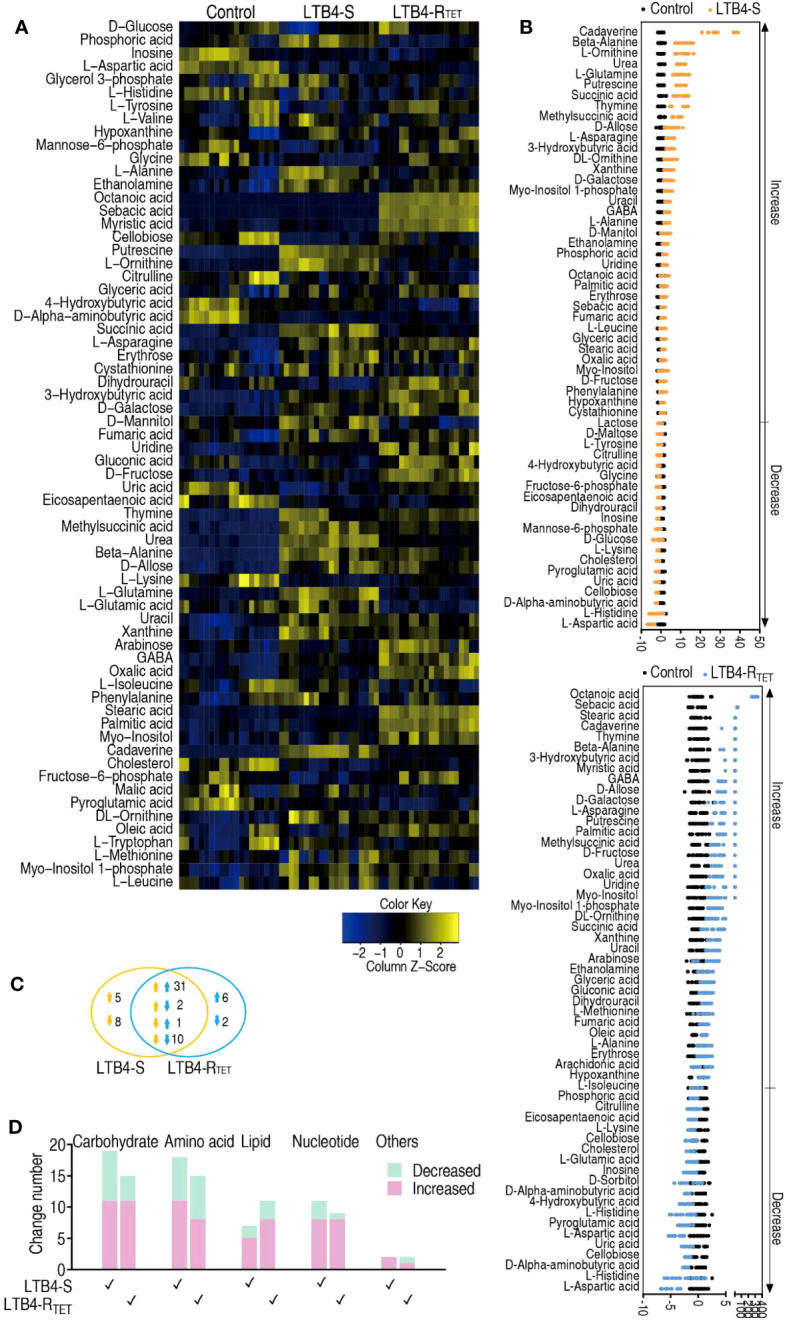
Metabolic profiles of differential metabolites. **(A)** Heat maps showing differentially expressed metabolites (row). Blue, downregulated; yellow, upregulated (see color scale). **(B)** Z-score plot showing the deviations of differential metabolites. **(C)** Venn diagram for the overlapping and unique metabolites with differential abundances between the two strains. Downward facing arrow, decreased metabolites; Upward facing arrow, increased metabolites. **(D)** Categories of metabolites with differential expression in the two strains.

To explore LTB4-R_TET_- and LTB4-S-induced metabolic features, a two-sided Mann–Whitney U test was used to identify differential abundance of metabolites in the two groups compared to control animals. We identified a total of 65 metabolites showing differential abundance (**Figure 3A**). Specifically, LTB4-S- and LTB4-R_TET_-infected fish had 57 and 52 differentially expressed metabolites, respectively. A Z-score calculation was used to display deviations between a value and the mean (**Figure 3B**). Among the 57 differentially expressed metabolites in LTB4-S-infected fish, 37 were upregulated while 20 were downregulated. Similarly, in LTB4-R_TET_- infected zebrafish, out of the 52 differentially abundant metabolites, 37 were also upregulated while 15 were downregulated. The top five downregulated metabolites in both groups overlapped, with ranking from the lowest to highest abundance of these five metabolites being: aspartate < histidine < aminobutyric acid < cellobiose < uric acid (**Figure 3B**). In total, we identified 44 overlapping metabolites between the two infection models as well as 13 (5 up- and 8 downregulated) and 8 (6 up- and 2 downregulated) unique changes in LTB4-S and LTB4-R_TET_ infected fish, respectively. Among the 44 overlapping metabolites, 41 displayed the same change with 31 upregulations and 10 downregulations (**Figure 3C**). An increased number of upregulated metabolites were identified compared to downregulated metabolites in carbohydrate, amino acid, lipid and nucleotide groups of both infections (**Figure 3D**). These data suggest that a similar metabolic shift is characterized between infections caused by LTB4-S and LTB4-R_TET_.

### 3.4 Metabolic pathway enrichment in zebrafish infected with LTB4-R_TET_ and LTB4-S

**Figure 4:**
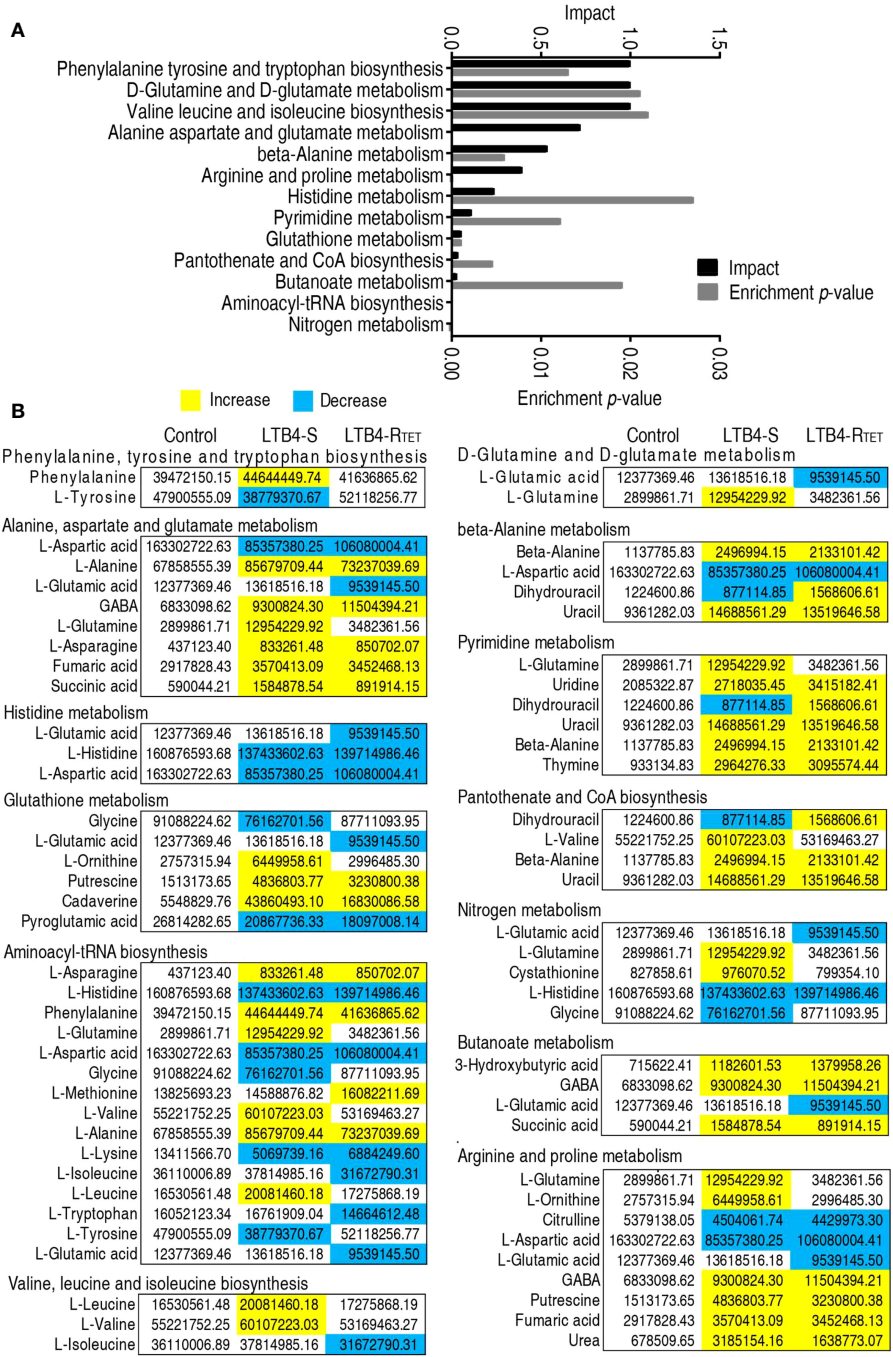
Metabolic pathway enrichment. **(A)** Enrichment of metabolic pathway by the metabolites with differential abundances. **(B)** Integrative analysis of the metabolites in enriched pathways. Yellow, upregulated metabolites; Blue, downregulated metabolites.

Having data on metabolic pathway alterations provides information that aids our understanding of key changes in the metabolic state. Thus, differential metabolite expression was analyzed, leading to data showing the enrichment of 13 metabolic pathways. Among them, phenylalanine, tyrosine, and tryptophan; D-glutamine and D-glutamate metabolism; valine, leucine, and isoleucine biosynthesis; alanine, aspartate, and glutamate metabolism; beta-alanine metabolism, and arginine and proline metabolism were ranked as the top six enriched metabolic pathways (**Figure 4A**). With the exception of histidine metabolism, where the abundance of all metabolites (aspartate, histidine, and glutamic acid) was reduced, the other metabolic pathways exhibited both increased and decreased metabolite expression. We hypothesized that the downregulated metabolites were associated with the dying animals. Therefore, we became interested in identifying a key depressed metabolite that overlapped between LTB4-S and LTB4-R_TET_-infected animals as a potential reprogramming metabolite. A total of five metabolites overlapped among metabolites in these enriched metabolic pathways, including aspartate, histidine, pyroglutamic acid, lysine, and citrulline (**Figure 4B**).

### 3.5 Biomarker identification in zebrafish infected with LTB4-R_TET_ and LTB4-S

**Figure 5:**
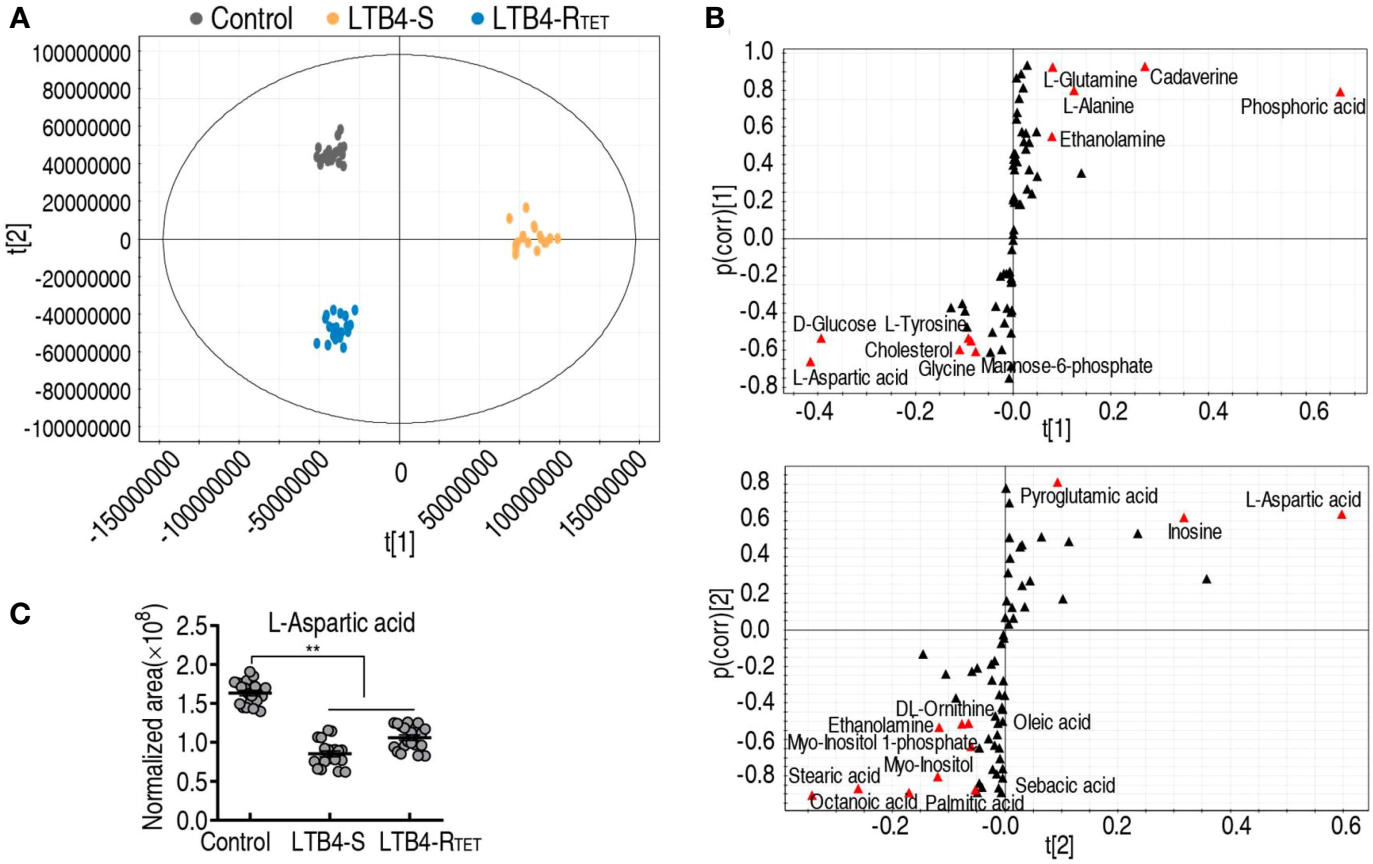
Identification of biomarkers. **(A)** PCA of uninfected control animals, LTB4-S, and LTB4-R_TET_. The technical replicates of samples are showed by dots in the plot. **(B)** S-plot is generated from OPLS-DA. Uninfected control animals and LTB4-R_TET_ are differentiated from LTB4-S by using predictive component t[1] and correlation p(corr)[1]. Uninfected control animals and the deviations of LTB4-S are separated from LTB4-R_TET_ by using predictive component t[2] and correlation p(corr)[2]. Ttriangle, metabolites; marked in red, candidate biomarkers. **(C)** Scatter diagram of crucial biomarkers in data **(B)**. **, P, 0.01.

Pattern recognition is an efficient method to identify biomarkers in metabolomics analyses. Thus, orthogonal partial least square discriminant analysis (OPLS-DA) was employed to recognize the sample patterns of differential metabolomes. Here, t[1] was separated control and LTB4-R_TET_ animals from the LTB4-S group, while t[2] differentiated control animals from the LTB4-R_TET_ group and deviation of LTB4-S (**Figure 5A**). An S-plot was used to identify discriminating variables with cutoff values of ≤0.05 and ≥0.5 for the absolute value of covariance p and correlation p(corr), respectively, as biomarkers. The data showed that t[1] identified downregulated aspartate, glucose, tyrosine, glycine, cholesterol, mannose-6-phosphate, and upregulated ethanolamine, alanine, glutamine, cadaverine, phosphoric acid as biomarkers. In contrast, t[2] determined downregulated aspartate, inosine, pyroglutamic acid, and upregulated stearic acid, octanoic acid, palmitic acid, stearic acid, myo-inositol, myo-inositol 1 phosphate, ethanolamine, ornithinem, oleid acid as biomarkers (**Figure 5B**). Among them, downregulated aspartate was found to overlap (**Figure 5C**), thereby highlighting aspartate as the most promising biomarker related to zebrafish death in the two infection models.

### 3.6 Aspartate protects zebrafish against infection caused by LTB4-S and LTB4-R_TET_

**Figure 6:**
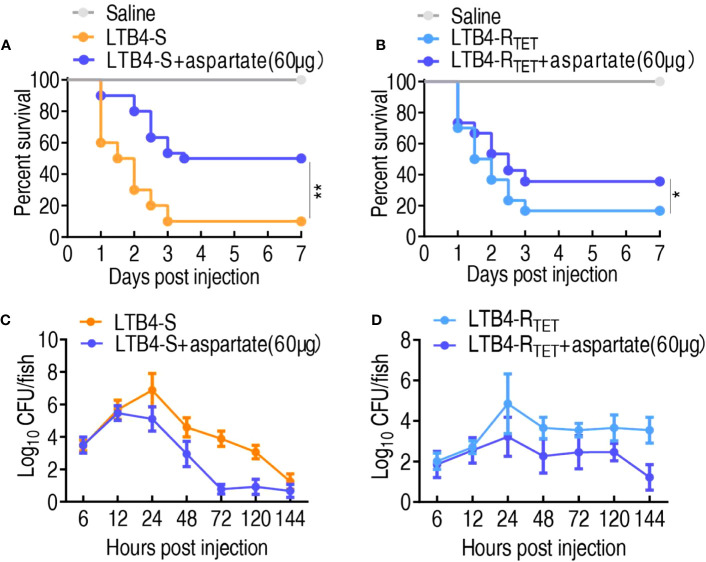
Role of aspartate in a zebrafish model. **(A, B)** Survival of zebrafish infected with and without aspartate and then infected with LTB4-S **(A)** or LTB4-R_TET_ **(B)**. **(C, D)** Bacterial load in the internal organs of zebrafish injected with and without aspartate and then infected with LTB4-S **(C)** or LTB4-R_TET_ **(D)**. *p < 0.05, **p < 0.01.

The above finding that the downregulated aspartate is related to zebrafish death suggested that the upregulation of aspartate may protect zebrafish against both LTB4-S and LTB4-R_TET_ infections. To test this idea, aspartate was complemented in zebrafish which were then infected with LTB4-S and LTB4-R_TET_. Aspartate improved zebrafish survival from 10% and 16.7% to 50% and 36.7%, respectively (**Figures 6A, B**). Meanwhile, bacterial load was also used as an important index to evaluate the role of aspartate. Similar to the observed changes in survival, exogenous aspartate promoted the elimination of LTB4-S and LTB4-R_TET_. Specifically, exogenous aspartate reduced LTB4-S and LTB4-R_TET_ by at least 2- and 4-fold after 24 h, respectively (**Figures 6C, D**). Therefore, aspartate is effective at protecting zebrafish from LTB4-S and LTB4-R_TET_ infection.

### 3.7 Aspartate promotes nitric oxide expression to eliminate LTB4-S and LTB4-R_TET_

**Figure 7:**
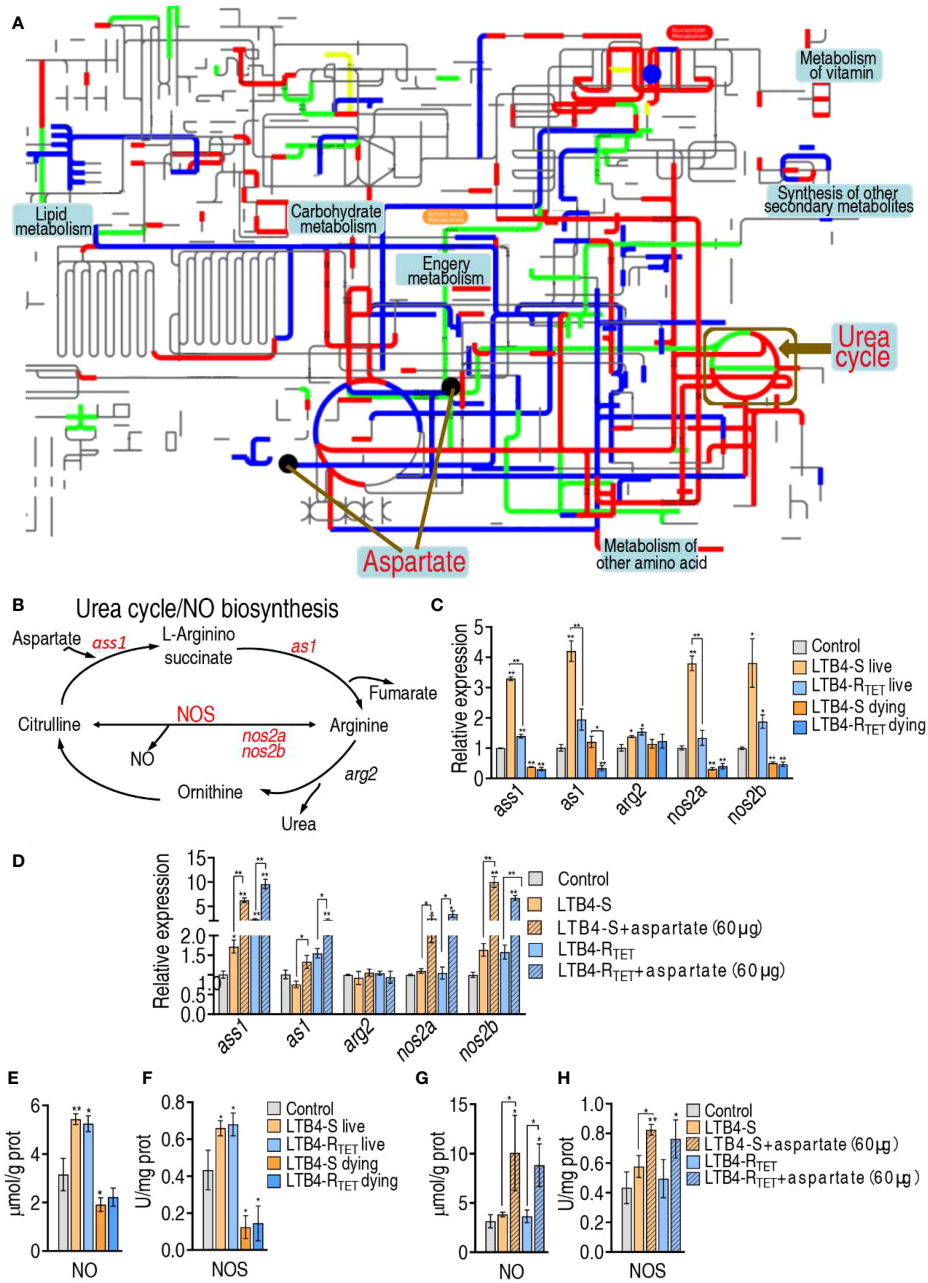
Role of NO in the aspartate potentiation. **(A)** iPath analysis for global metabolic changes. **(B)** Urea cycle and NO biosynthesis. **(C, D)** qRT-PCR for NO generation genes expression in control, LTB4-S, and LTB4-R_TET_ **(C)** or plus aspartate **(D)**. **(E)** NO level in control, LTB4-S, and LTB4-R_TET_ animal groups. **(F)** NOS activity in control, LTB4-S, and LTB4-R_TET_ animal groups. **(G)** NO level in control, LTB4-S, and LTB4-R_TET_ animal groups in the presence of aspartate. **(H)** NOS activity in control, LTB4-S, and LTB4-R_TET_ animal groups in the presence of aspartate. *, P , 0.05; **, P , 0.01.

Aspartate has multiple metabolic pathways. To identify the affected pathway in LTB4-S and LTB4-R_TET_-infected zebrafish, iPath was employed to analyze differential abundances of metabolites (**Figure 7A**). LTB4-S and LTB4-R_TET_ infections had their own specific differential pathways, but overlapping pathways were also identified. We were interested in the overlapping metabolic pathways that were involved in aspartate regulation. Our data showed that aspartate is involved in four overlapping metabolic pathways, where the metabolic flux from aspartate to the urea cycle is fully affected (**Figure 7A**). Aspartate is a source for the urea cycle, where NO is synthesized from an L-arginine by NOS (**Figure 7B**). Next, we performed qRT-PCR to measure expression of genes encoding the cycle in surviving and dying LTB4-S- and LTB4-R_TET_-infected zebrafish. Decreased and increased expression of *ass1, as1*, *nos2a*, and *nos2b* was measured in dying and surviving animals, respectively, compared to control animals. Interestingly, *arg2* expression remained unchanged in the LTB4-S infection model. Notably, expression of the four genes was higher in animals infected with LTB4-S compared to LTB4-R_TET_ (**Figure 7C**). When aspartate was complemented, higher expression of the four genes was measured (**Figure 7D**). Further, the activity of NOS and NO was lower in the dying animals but higher in the surviving animals compared to the control group (close to significant difference in NOS of the dying fish caused by LTB4-R_TET_) (**Figures 7E, F**). However, aspartate complement promoted NOS activity and NO level even if zebrafish were challenged by LTB4-S and LTB4-R_TET_ (**Figures 7G, H**). Taken together, these data confirm that aspartate promotes NO production.

### 3.8 Sodium nitroprusside promotes zebrafish to eliminate LTB4-S and LTB4-R_TET_

**Figure 8:**
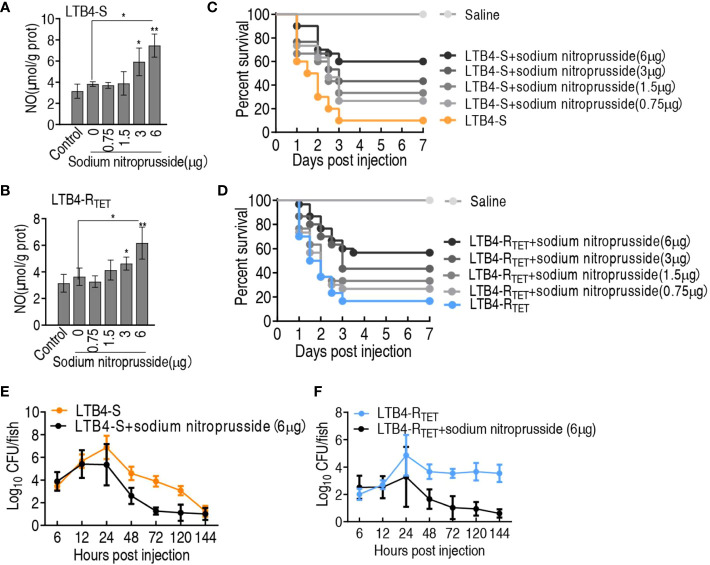
Role of sodium nitroprusside in the aspartate potentiation. **(A, B)** NO level of zebrafish in the indicated sodium nitroprusside plus LTB4-S **(A)** or LTB4-R_TET_ **(B)**. **(C, D)** Survival of zebrafish infected with LTB4-S **(C)** or LTB4-R_TET_ **(D)** and with and without the indicated sodium nitroprusside. **(E, F)** Bacterial load in internal organs of zebrafish infected with LTB4-S **(E)** or LTB4-R_TET_ **(F)** and with and without sodium nitroprusside. *p < 0.05, **p < 0.01.

To demonstrate that NO plays a role in the elimination of LTB4-S and LTB4-R_TET_, NO donor sodium nitroprusside was used to test protection against infection caused by LTB4-S and LTB4-R_TET_. NO was elevated during treatment with 3–6 μg sodium nitroprusside (**Figures 8A, B**). Sodium nitroprusside increased zebrafish survival in both infection models in a dose-dependent manner leading to survival rates of 55.5% and 48%, respectively, following treatment with 6 μg sodium nitroprusside (**Figures 8C, D**). In addition, we found that sodium nitroprusside consistently caused a reduction in bacterial load in both experimental groups. Specifically, sodium nitroprusside reduced LTB4-S and LTB4-R_TET_ by at least 2- and 3-fold after 24 h, respectively (**Figures 8E, F**). These results indicate that NO plays an important role in protecting zebrafish from LTB4-S and LTB4-R_TET_ infection.

## 4 Discussion

Antibiotic-free therapy against bacterial infection is recommended as this strategy avoids antibiotic-related shortcomings (32, 33). Among the antibiotic-free therapy, reprogramming of the metabolome/metabolic state is an effective approach to restore host protection from microbes. In the present study, we used this approach to explore the potential of using a single reprogramming metabolite to combat both antibiotic-sensitive and -resistant *E. tarda*. In this way, we have identified aspartate as the ideal reprogramming metabolite and we have shown that it is downregulated in both LTB4-S- and LTB4-R_TET_-infected dying zebrafish. Moreover, exogenous aspartate protects zebrafish to eliminate LTB4-S and LTB4-R_TET_, thereby improving survival. Mechanistically, exogenous aspartate-mediated metabolic flux promotes NO biosynthesis to induce its protective effect, which is further validated by NO donor sodium nitroprusside that also promotes the elimination of both LTB4-S and LTB4-R_TET_ in zebrafish to increase the survival rate of these infected animals as exogenous aspartate does. These results suggest that aspartate-mediated NO may be an effective approach for combating both antibiotic-sensitive and -resistant *E. tarda*, providing a previously unknown antibiotic-free therapeutic modality to eliminate *E. tarda* with both antibiotic sensitivities.

Reprogramming of the metabolome/metabolic state has previously been adopted to promote antibiotic killing efficacy (34–37), as well as to protect hosts against bacterial infection (20, 38–40). In the latter, reprogramming metabolites are identified from the comparison between control and surviving infected animals (41, 42). In a change from this, the present study explores whether reprogramming metabolites may be identified from the comparison between control and dying animals in *E. tarda* infection models. Our robust proof of concept study indicates that biomarkers identified from dying animals can be used as reprogramming metabolites. These data will be helpful in utilizing dying instead of surviving animals to identify reprogramming metabolites for low-antibiotic and antibiotic-free strategies against bacterial pathogens, which is especially important for animals with high economic value.

Thus, we have identified aspartate as the most promising biomarker, and demonstrated its downregulation in zebrafish infected with LTB4-S or LTB4-R_TET_ compared to control fish. Exogenous aspartate was also shown to be an ideal reprogramming metabolite to combat both LTB4-S and LTB4-R_TET_ infection. Based on aspartate metabolism, it is logical to hypothesize that high doses of aspartate promote NO generation, given aspartate acts as a source for its biosynthesis. On the other hand, NO is a key gas messenger in the pathogenesis of inflammation, where it links innate and adaptive immunity (43, 44). NO-mediated elimination of bacterial pathogens has previously been documented (45, 46), but there are no specific reports of NO-mediated elimination to *E. tarda*. Therefore, the present study focused on answering two questions: does exogenous aspartate promotes NO biosynthesis and does NO combat antibiotic-sensitive and -resistant *E. tarda*? Our results show that high doses of aspartate elevate NO biosynthesis and that sodium nitroprusside-induced elevation of NO also protects zebrafish against LTB4-S and LTB4-R_TET_ infection. Therefore, downregulation of NO due to bacterial infection is a cause of zebrafish failure to eliminate LTB4-S and LTB4-R_TET_ pathogens. Therefore, indirect complementation of NO by aspartate metabolic reprogramming or directly by sodium nitroprusside restores protection against LTB4-S and LTB4-R_TET_ infection.

## 5 Conclusion

Herein, we show that aspartate is depressed in dying zebrafish infected with LTB4-S and LTB4-R_TET_ and we have validated its role as a reprogramming metabolite. Exogenous aspartate restores zebrafish protection against LTB4-S and LTB4-R_TET_ infection by increasing NO synthesis. These results suggest that reprogramming metabolites can be identified from both surviving and dying animals. Promoting NO production is an important method for promoting aspartate-mediated elimination of pathogenic bacteria. Because metabolites have multiple metabolic pathways to produce different effect products, only by identifying the metabolic pathways that play a role can we reveal underlying mechanisms and develop reversal strategies.

## Data availability statement

The original contributions presented in the study are included in the article/supplementary material. Further inquiries can be directed to the corresponding author.

## Ethics statement

The animal study was approved by the Institutional Animal Care and Use Committee of Sun Yat-sen University (Approval No. SYSU-IACUC- 2020-B1267). The study was conducted in accordance with the local legislation and institutional requirements.

## Author contributions

HL: Conceptualization, Writing – original draft, Writing – review & editing. JX: Data curation, Investigation, Methodology, Writing – original draft. M-YL: Investigation, Methodology, Writing – original draft. 
